# Appointment Length with Patients in Medical Consultations in Bangladesh: A Hospital-Based Cross-Sectional Study

**DOI:** 10.3390/healthcare9091164

**Published:** 2021-09-05

**Authors:** Madhab Chandra Das, Muhammad Zakaria, Feng Cheng, Junfang Xu

**Affiliations:** 1 Department of Communication and Journalism, University of Chittagong, Chittagong 4331, Bangladesh; madhabdas@cu.ac.bd (M.C.D.); zakaria@cu.ac.bd (M.Z.); 2 Vanke School of Public Health, Tsinghua University, Beijing 100084, China; fcheng@mail.tsinghua.edu.cn; 3 Institute for Healthy China, Tsinghua University, Beijing 100084, China; 4 Center for Health Policy Studies, School of Public Health, Zhejiang University School of Medicine, Hangzhou 310058, China

**Keywords:** inequalities, medical consultation, consultation length, appointment length, patient-centered behavior, Bangladesh

## Abstract

In medical consultations, the length of the visit has a significant impact on the quality of care. It is significantly associated with a better quality of treatment and better health outcomes. In this study, we analyzed doctors’ consultation length with patients and associated factors in Bangladesh. A cross-sectional survey was conducted among the patients (N = 763) who visited the doctors in six district/upazila (sub-district) hospitals in the Chittagong Hill Tracts (CHT) area. Linear regression analyses were performed to identify the determining factors associated with the length of doctors’ appointments with patients. Data were analyzed using IBM SPSS version 24.0. Among the patients, 319 (41.8%) were female and 688 (90.2%) lived in rural/suburban areas. This study revealed that the average length of medical consultations was 9.10 min. Additionally, our findings illustrated that doctors’ patient-centered communication behavior (β = 0.23, *p* < 0.001) appeared to be the strongest predictor of longer visit length. It was also found that patients’ higher education level (β = 0.10, *p* = 0.006), having adequate knowledge about the health problem (β = 0.13, *p* < 0.001), follow-up visits (β = 0.13, *p* < 0.001), and the presence of female doctors (β = 0.19, *p* < 0.001) were significantly associated with longer interview times between doctors and patients in primary care settings. Given that doctors’ patient-centered communication behavior appears to play the most important role, this study suggests that practicing professionalism in medical consultations, developing effective communication skills and increasing awareness of sociodemographic discrepancies are important to ensure longer appointment lengths and better health outcomes of patients, regardless their sociodemographic and socioeconomic status.

## 1. Introduction

Longer consultation times are considered to provide an important means of effective health communication between doctors and patients. Moreover, it is also associated with better quality of treatment and better health outcomes [1]. This is because the longer the appointment lasts, the more likely patients would be to participate, resulting in a more reliable outcome for the consultation [2,3,4]. Studies have also shown that an increased consultation time can contribute to enhanced patient safety, medication adherence, decreased costs of medical malpractice, and increased patient satisfaction across the healthcare sector [5,6].

However, only a small percentage of patients were allowed to finish the initial descriptions of their concerns [7,8]. General physicians argued that 10-minute visits are unsustainable and that primary care appointments should last at least 15 min, including examinations and check-ups [9]. According to a survey conducted by the British Medical Association, 92 percent of 15,560 general physicians agreed that 10 min is insufficient for primary care consultations [9].

The length of a consultation is affected by many factors, including the characteristics of the doctor, patients, and clinic type. Orton and Gray [10] found that doctors’ gender, experience, degree of emotional exhaustion, and patient-centeredness were associated with the consultation length in general practice. Furthermore, Wiggers and Sanson-Fisher [11] argued that the location of the medical settings affects the length and urban consultations are longer than those of rural settings. According to a study conducted by Britt et al. [12], older female patients with higher socioeconomic status also tended to have longer consultations. However, there has been no research focusing on appointment length and identifying its factors in Bangladesh. Against this background, we aim to analyze the appointment duration for doctors and patients in primary care medical consultations and identify the influencing factors in Bangladesh. This study could provide evidence for health policymakers and program planners to design interventions to improve communication between doctors and patients by increasing the appointment length appropriately.

## 2. Methods

### 2.1. Study Design and Sample Size

A hospital-based cross-sectional design was used to collect data. It was conducted in six districts and upazilas (sub-districts) hospitals in the Chittagong Hill Tracts (CHT) area of Bangladesh. Three district hospitals (Rangamati, Bandarban, and Khagrachori) in the CHT region were chosen for hospital patients because they served a more diverse population than that of upazilas. The lottery approach was used to choose three upazilas from three districts to obtain data from the Upazila Health Complexes (UHC). Selected UHCs are the Rajasthali Upazila Health Complex from Rangamati district, the Rowangchhari Upazila Health Complex from Bandarban district, the Lakshmichari Upazila Health Complex from Khagrachori district.

The sample size was determined using a single-population proportion formula considering the following assumptions: *p* = 50%, significance level 5% (α = 0.05), Z $\frac{\alpha }{2}$ = 1.96, margin of error 3% (d = 0.05), a design effect of two (as stratified multistage sampling is used) and 10% non-response rate.

$$\begin{array}{ll} N & =\frac{{\left(Z\frac{\alpha }{2}\right)}^{2}P\left(1-P\right)}{d^{2}}\\ & =\frac{1.96^{2}\times 0.50\times 0.50}{0.05^{2}}\\ & N=770 \end{array}$$

A total of 770 patients participated in the study, and 763 respondents filled up the questionnaire completely indicating a 99.1% response rate.

### 2.2. Data Collection Tool and Procedures

Data were collected using a structured, facilitator-administered, and post-consultation questionnaire prepared in Bengali. The questionnaire was divided into four sections, i.e., (a) patients’ sociodemographic and socioeconomic characteristics, for example, education, occupation, age, sex, ethnicity, monthly family income, area of residence, marital status; (b) patients’ cognitive and predisposing variables, such as type of visit, perception of having an adequate idea about the disease, expression of anxiety to the doctor, giving an opinion about medication; (c) doctors’ predisposing variables including gender, appointment length; (d) doctors’ patient-centered communication behavior. This behavioral construct was developed by Wachira et al. [13].

### 2.3. Validity and Reliability of the Instrument

The content validity of the questionnaire was reviewed by three experts who had worked in the same field in order to establish the relevance of the questionnaire items to the study aims. The experts reviewed the questionnaire separately. The reviewers’ identities were not revealed to each other aside from the researcher. Some changes were made in the questionnaire based on experts’ recommendations. The internal consistency of the doctors’ patient-centered communication behavior was also measured. Cronbach’s alpha (α) was 764, confirming that the instrument is valid for this particular sample.

### 2.4. Data Quality Management

The questionnaire was pre-tested among 40 patients outside the study area, and then it was examined for suitable wording, content consistency, and whether the directions elicited corresponded to the responses. Two days of intensive training were given to six data collectors selected from the University of Chittagong, Faculty of Social Sciences, before data collection. The training focused on the techniques of approaching the study participants, the purpose of the study and the variables of the questionnaire, and issues related to doctor-patient medical consultation.

### 2.5. Data Processing and Analysis

The data were coded and entered into IBM SPSS version 24, where they were reviewed and cleaned for completeness and codingbefore being analyzed. The independent-samples *t*-test and Pearson correlations were calculated to compare the mean appointment length with the independent variables and to see if the difference was statistically significant or not. Most of the variables were fitted to the bivariate analysis. Then, all variables having a *p*-value ≤  0.05 in the bivariate analysis were further entered into the hierarchical linear regression model. The multicollinearity was checked. In hierarchical regression, step 1 assessed the effects of patients’ socio-demographic variables on appointment length. Step 2 explored the effects of patients’ socio-demographic, cognitive, and predisposing factors, whereas step 3 examined the effects of patients’ socio-demographic, cognitive and predisposing, and doctors’ predisposing factors. In the model summary, the ANOVA values (*p* < 0.001) of each step associated with appointment length demonstrated that our hierarchical regression model performed well and would be a good predictor of the main outcome variables. The *R^2^* value of each step changed considerably, and *F* changes were also statistically significant (*p* < 0.001). Variables having *p*-values < 0.05 in the regression analysis were taken as significant predictors of longer appointment length.

## 3. Results

### 3.1. Sociodemographic and Socioeconomic Characteristics of Patients

Table 1 displays the socio-demographic characteristics of the patients. Data of 763 patients were analyzed. Of them, 208 (27.3%) had no formal education, whereas only 179 (23.5%) had an education level above a secondary school certificate. In regard to the patients’ ethnic identity, 345 (45.2%) were *Bengali* and 418 (54.8%) belonged to an ethnic minority group. Among the patients, 319 (41.8%) were female and 688 (90.2%) lived in rural/suburban areas. Among them, 163 (21.4%) were housewives or jobless, whereas 198 (26%) were involved in agriculture. In addition, the respondents’ average monthly household income was BDT 16,138, and 487 (63.8%) were married.

### 3.2. Appointment Length and Relationships with Various Characterizes

**Figure 1:**
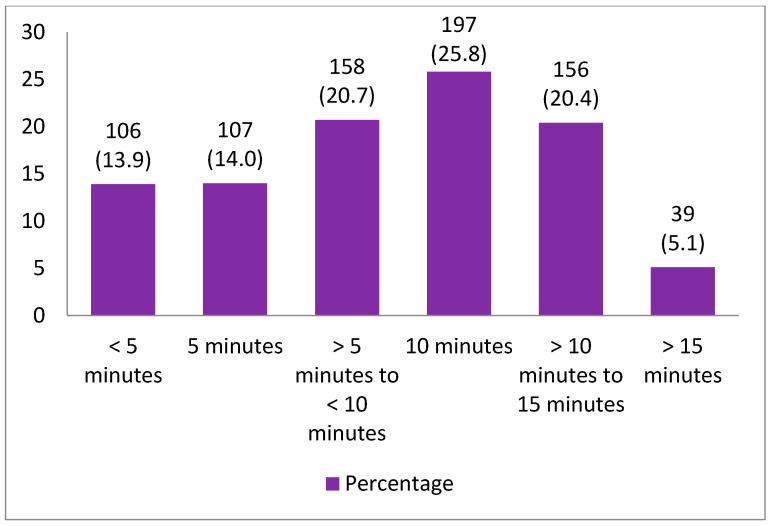
Percentage distribution of appointment length of medical consultations.

**Figure 2:**
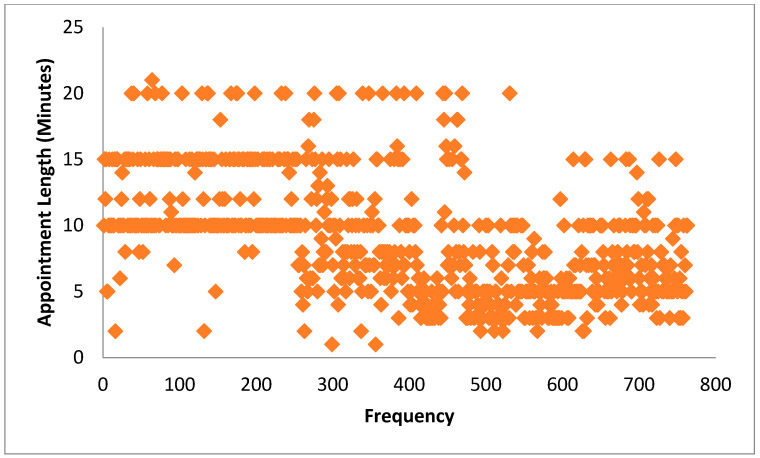
Frequency distribution of appointment length of medical consultations.

Figure 1 and Figure 2 display the percentages and frequency distributions of doctors’ appointment lengths with patients. The average consultation length with patients was 9.10 min (SD ±4.44). Of the study participants, 196 (25.8%) had an appointment length of 10 min in their medical consultation, followed by 158 (20.7%) whose appointments ranged lasted from five minutes to 10 min, 156 (20.4%) from 10 min to 15 min, 107 (14%) that lasted five minutes, 106 (13.9%) that lasted below five minutes and the remaining 39 (5.1%) had appointments longer than 15 min. Table 2 illustrates that the patients’ education (*t* = 5.14, *p* < 0.001), ethnicity (*t* = 6.80, *p* < 0.001), and monthly family income (*r* = 0.20, *p* < 0.0.001) were significantly linked with longer appointment lengths in primary care medical consultations. The results also showed that the patients’ visit type (*t* = 5.24, *p* < 0.001), their perception of having an adequate idea of their disease (*t* = 6.34, *p* < 0.001), giving opinions about medication (*t* = 3.22, p = 0.001), the gender of the doctors (*t* = 4.39, *p* < 0.001), and doctors’ patient-centered communication behavior (*r* = 0.37, *p* < 0.001) were positively related with longer appointment length in medical consultations.

### 3.3. Predictors of Longer Appointment Length in the Primary Medical Consultations

Table 3 shows the factors influencing longer appointment length among the study participants. The hierarchical multiple regression results reported in Table 3 reveal that in step 1 (patients’ socio-demographic variables), three predictors of longer appointment length—for example, patients’ education (β = 0.16, *t* = 4.72, *p* < 0.001), being Bengali (β = 0.21, *t* = 5.95, *p* < 0.001), and family income (β = 0.12, *t* = 3.42, *p* = 0.001)—contributed significantly to the regression model (F = 29.47, df = 3/759, *p* < 0.001) and accounted for 10% of variations in longer appointment length among the study participants.

Adding the other three predictors in step 2 explained an additional 5% of the variations in the appointment length, above and beyond the effects of the predictors in step 1. In step 2, among the six predictors of longer appointment length in medical consultations—education (β = 0.14, *t* = 4.01, *p* < 0.001), being Bengali (β = 0.18, *t* = 5.10, *p* < 0.001), family income (β = 0.11, *t* = 3.00, *p* = 0.003), having an adequate idea of their disease (β = 0.15, *t* = 4.21, *p* < 0.001), follow-up visit (β = 0.15, *t* = 4.36, *p* < 0.001), and giving their opinion about their medication (β = 0.07, *t* = 2.15, *p* = 0.032)—contributed significantly to the regression model (*F* = 23.49, df = 3/756, *p* < 0.001) and accounted for 15% of variations in appointment length among the study participants.

Introducing the other two predictors in step 3 explained an additional 5% of variations in appointment length, above and beyond the effects of the predictors in step 2. In step 3, among the eight predictors of appointment length, education (β = 0.10, *t* = 2.78, *p* = 0.006), family income (β = 0.08, *t* = 2.29, *p* = 0.02), having an adequate idea of their disease (β = 0.13, *t* = 3.84, *p* < 0.001), follow-up visit (β = 0.13, *t* = 4.02, *p* < 0.001), visiting a female doctor (β = 0.19, *t* = 3.61, *p* < 0.001), and doctors’ patient centered communication behavior (β = 0.23, *t* = 5.64, *p* < 0.001) contributed significantly to the regression model (*F* = 24.72, df = 2/754, *p* < 0.001) and accounted for 20% of variations in longer appointment length among the patients.

## 4. Discussion

The interview length between doctors and patients was not quite sufficient for one quarter of the patients who responded in this study. Almost 30% had a visit time of fewer than five minutes. However, the average consultation time in our study was much higher than previous studies, which reported that the average consultation time in a government hospital was 2.33 min [14], and 3.51 min has been reported in government facilities [15]. Insufficient consultation time has been previously noted as one of the significant factors contributing to patient dissatisfaction in Bangladesh [14,15]. Indeed, patients’ satisfaction with the doctors’ services is not very good in Bangladesh [16]. The reasons for this may include a lack of professionalism among health care professionals, the dearth of accountability, long queues of patients, doctors’ tendencies to push patients into private hospitals, and doctors’ proneness to spend more time in private chambers with patients who pay a high fee, which can also result in doctors’ spending insufficient time with patients in government settings. Besides, because most of the country’s reputable doctors represent several hospitals, they are unable to give patients due time and attention. There is no recorded assessment of the quality of doctor care in Bangladesh, in both the public and private sectors, according to the World Bank [17].

In our study, we found that doctors provided educated patients with longer consultations, which may indicate that these patients generally have a better understanding of their healthcare options and treatment options. Generally, educated people are more conscious of their illnesses than those who are less educated. They appear to ask more questions, and talk longer than other patients [18]. However, in previous studies, doctors reported that less-educated patients have trouble properly voicing their issues. Even more often than not, they misinterpret doctors’ advice and ask the same questions repeatedly, dissuading doctors from spending more time with them. Our results are consistent with previous studies [13,19,20,21].

When it comes to the quality of healthcare, financial capacity is also important. Financially capable patients are more likely to pay their doctors’ bills on time. They also have stronger links to other affluent individuals. As a result, maintaining a positive working relationship with affluent people can help doctors to attract more patients and develop a loyal following. Higher-income patients are often treated with more regard due to socioeconomic prejudice than their lower-status counterparts. As a result, doctors often treat affluent patients with greater care and devote more time to them. Our findings in this regard are also in line with those of previous studies [12].

Having an adequate idea of disease was also a significant factor influencing the appointment length in our study. Patients with ample knowledge of their prior diseases and drugs may provide more detailed information, which assists the doctor in evaluating their issue and identifying the root cause. As a result of the increased patient involvement in the process, the conversation improves.

In comparison to the first visit, follow-up visits usually require deeper contact between doctors and patients. In the majority of cases, during follow-up visits, doctors usually ask more specific questions if they need more detail by checking the results of reports or comparing them to previous ones. This may lead to a more accurate diagnosis and a longer consultation period.

Female and male doctors’ practice styles varied greatly, and female doctors’ patients were usually satisfied with their care. In Bangladesh, there are still many social taboos around health issues. As a result, female patients are more likely to seek out female doctors to address their health problems. They assume that since the doctor is a woman, she will be able to better understand them. Since female doctors are the primary caregivers of most households, they are more likely to pay more attention to and consider their patients’ issues carefully [21,22]. This shared understanding between female doctors and patients may also be associated with longer appointment times. Furthermore, many female doctors also face discrimination due to Bangladesh’s patriarchal socio-economic conditions. They sometimes feel obligated to show their worth as dependable doctors. In addition, previous studies also showed that female doctors spent an average of 2 min, or 10%, more time with their patients than male doctors per visit [10,23,24], which are consistent with previous studies.

We also found that doctors’ patient-centered communication behavior had a significant impact on appointment length in medical consultations. If a doctor lacks effective communication skills, the patients are less likely to participate in the medical consultation. Patients, on the other hand, are more likely to get involved in expressing their concerns, asking questions and giving opinions about their treatment in a consultation in which the doctor provides supportive communication behaviors. Our results, consistently with previous studies, showed a correlation between appointment duration and doctors’ patient-centered behavior [2,3,4,10,25,26,27,28,29,30].

Our study has some limitations. First, this research was unable to obtain a clearer explanation and a more in-depth understanding of the issue, since no qualitative data collection tool was used. Moreover, we examined quantitative measures of doctor-patient medical consultations rather than qualitative measures of the issue. Secondly, social desirability may have influenced the participants’ responses, influencing the validity of the findings.

## 5. Conclusions

We found that doctors’ patient-centered communication behaviors were significantly associated with longer appointment lengths. Beyond simply spending more time with the patients, doctors could better perform quality-of-care suggestions by facilitating patient involvement and individual interaction. Therefore, it is important for doctors to practice professionalism in medical consultations, to develop effective communication skills and to increase their awareness of sociodemographic discrepancies to ensure longer appointment lengths, quality healthcare and better health outcomes of patients, regardless of their sociodemographic and socioeconomic status.

We did not investigate whether the variables examined in this study predict post-consultation outcomes, for instance, patient satisfaction, treatment adherence and health outcomes. Future studies should not only include outcomes but should also address the new empirical data on sociodemographic inequality, medical consultations, and satisfaction ratings, which could improve the conversations between the two counterparts. Further research should also consider specific organizational, interpersonal, cognitive, and cultural factors that account for variability related to demographic characteristics.

## Figures and Tables

**Table 1:** Demographic and socio-economic characteristics of study participants (N = 763).

Variables	Categories	Frequency	Percentage
Education of patients	No education	208	27.3
	Up to class 5	106	13.9
	Class > 5–8	120	15.7
	Class > 8–10	150	19.7
	Class > 10	179	23.5
Ethnicity	Bengali	345	45.2
	Ethnic minority	418	54.8
Gender	Female	319	41.8
	Male	444	58.2
Place of residence	Urban	75	9.8
	Rural	688	90.2
Profession	Housewife/no job	163	21.4
	Agriculture	198	26.0
	Student	218	28.6
	Labour/service	88	11.5
	Business	96	12.6
Family income (Mean, ± SD)	BDT 16,138 (± 15,019)	-	-
Marital status	Married	487	63.8
	Single/widow/divorced	276	36.2

Note: BDT = Bangladeshi Taka.

**Table 2:** Patients’ socio-demographic, cognitive and predisposing predictors and doctors’ predisposing predictors of longer appointment length in medical consultations.

Variables	Categories	Mean	SD	*t/r*	*p*
Education of patients	No education/<class 5	8.16	3.84	5.14 ^a^	<0.001
	Class > 5	9.76	4.71		
Ethnicity	Bengali	10.27	4.30	6.80 ^a^	<0.001
	Ethnic minority	8.13	4.33		
Gender	Female	8.84	4.72	1.36 ^a^	0.17
	Male	9.29	4.23		
Place of residence	Urban/sub-urban	9.07	4.36	−0.14 ^a^	0.89
	Rural	9.12	4.50		
Family income (BDT)		-	-	0.20 ^b^	<0.001
Type of visit	Follow up visit	10.35	4.60	5.24 ^a^	<0.001
	First visit	8.55	4.26		
Perception of having an adequate idea about the disease	Yes	10.86	4.51	6.34 ^a^	<0.001
	No	8.54	4.28		
Expression of anxiety to doctor	Yes	9.07	4.50	−0.62 ^a^	0.54
	No	9.35	3.98		
Giving opinion about medication	Yes	9.48	4.50	3.22 ^a^	0.001
	No	8.41	4.27		
Gender of doctor	Female	10.25	4.42	4.39 ^a^	<0.001
	Male	8.68	4.38		
Patient-centered Communication		-	-	0.37 ^b^	<0.001

Note. ^a^ Two-tailed *t*-test; ^b^ Pearson correlation.

**Table 3:** Multiple linear regression analysis showing factors associated with longer appointment length in the medical consultation with the model summary ^d^.

Variables	Model 1 ^a^		Model 2 ^b^		Model 3 ^c^	
	β	*t*	β	*t*	β	*t*
Education of patients ^⸸^	0.16 ***	4.72	0.14 ***	4.01	0.10 **	2.78
Ethnicity ^⸷^	0.21 ***	5.95	0.18 ***	5.10	0.05	1.34
Family income ^§^	0.12 **	3.42	0.11 **	3.00	0.08 *	2.29
Adequate idea of their disease ^‡^			0.15 ***	4.21	0.13 ***	3.84
Type of visit ^¶^			0.15 ***	4.36	0.13 ***	4.02
Giving opinion about medication ^‡^			0.07 *	2.15	0.03	0.98
Gender of doctor ^∫^					0.19 ***	3.61
Communication score ^§^					0.23 ***	5.64
Adjusted *R^2^*	10%		15%		20%	
*SE* of the estimates	4.21		4.10		3.98	
*R^2^* Change	10%		5%		5%	
*F* Change	29.47		15.79		24.12	
*df1*	3		3		2	
*df2*	759		756		754	
Sig. *F* Change	<0.001		<0.001		<0.001	
ANOVA	*F* = 29.47, *p* < 0.001		*F* = 23.49, *p* < 0.001		*F* = 24.72, *p* < 0.001	

^⸸^ 1 = no education/< class 5, 2 = class > 5; ^⸷^ 1 = ethnic minority, 2 = Bengali; ^§^ continuous variable; ^¶^ 1 = first visit, 2 = follow-up visit; ^‡^ 1 = no, 2 = yes; ^∫^ 1 = male, 2 = female. Note: * *p* < 0.05, ** *p* < 0.01, *** *p* < 0.001; ANOVA = one-way analysis of variance. ^a^ Socio-demographic predictors. ^b^ Patients’ socio-demographic, cognitive, and predisposing predictors. ^c^ Patients’ socio-demographic, cognitive and predisposing predictors and doctors’ predisposing predictors. ^d^ Dependent variable: appointment length.

## Data Availability

All of the primary data have been included in the results. Additional materials with details may be obtained from the corresponding author.
